# An Undergraduate Chemistry Experiment Integrating Theoretical and Practical Aspects of Hypervalent Iodine(I) Compounds

**DOI:** 10.1021/acs.jchemed.5c01160

**Published:** 2026-02-27

**Authors:** Vladimir L. Kolesnichenko, Galina Z. Goloverda

**Affiliations:** Chemistry Department, 5785 Xavier University of Louisiana, 1 Drexel Dr., New Orleans, Louisiana 70125, United States

**Keywords:** Undergraduate Chemistry Experiment, Undergraduate Synthesis Lab, Hypervalent Elements, Polyhalide Anions, Interhalogen Adducts, Chloroiodate Synthesis, Chloroiodate Spectra, Electrophilic Iodination

## Abstract

A simple reaction sequence has been developed to produce iodine chloride-pyridine (PyICl) and pyridinium dichloroiodate (PyHICl_2_), convenient iodinating agents, in a high yield. Our approach is safe and simple to handle, avoiding the use of elemental chlorine or hazardous manipulations. Designed for an upper-level undergraduate hybrid lecture/lab chemistry course, the experiment integrates key concepts from both inorganic and organic chemistry. The pre-experiment lecture explains the connections between molecular structure, reactivity trends, and reaction types. Over three 4 h lab sessions, students carry out a sequence of quantitative transformations and characterize each intermediate and the final product using ESI mass spectrometry, as well as ^1^H and ^13^C NMR spectroscopy. They also apply the PyICl adduct for electrophilic iodination of salicylic acid. This experiment reinforces theoretical knowledge while providing hands-on experience in inorganic and organic synthesis and instrumental analysis. This material, along with its educational value, can also be useful to practical benchtop chemists working in the research and development sector.

## Graphical abstract

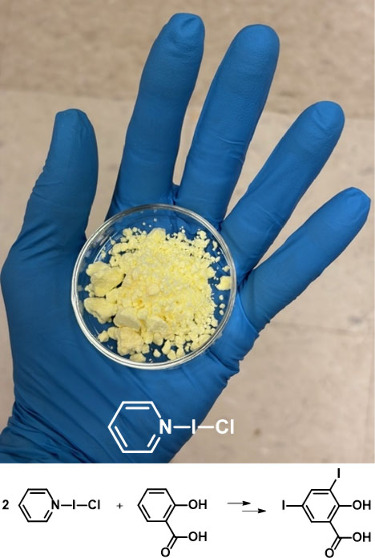

## Introduction

Many undergraduate students struggle to connect the knowledge acquired in different chemistry courses. For example, students in organic chemistry often overlook the nature of inorganic reagents used in synthesis, while those studying inorganic chemistry may not consider the function of inorganic reagents in organic synthesis. This disconnect typically persists until students encounter organometallic chemistry and catalysis in advanced-level courses. The aim of the present work is to help alleviate this gap in understanding.

Halogens, as representative p-block elements, are among the most compelling groups, providing rich opportunities for both theoretical and practical exploration. Inorganic and organic halides represent some of the most important classes of compounds in both inorganic and organic chemistry, with significance spanning laboratory research, industrial applications, and theoretical frameworks. Fluorinated and chlorinated polymers are widely used as specialty and construction materials. The importance of halides as intermediates in organic synthesis cannot be overstated.

From a theoretical standpoint, halogens provide classical examples for exploring chemical bonding, ranging from more or less polar covalent bonding in simple halides to bonding in “hypervalent” halogen compounds in higher oxidation states. Halogen-containing compounds are frequently used to introduce students to the Valence Shell Electron Pair Repulsion (VSEPR) model and the Molecular Orbital (MO) theory. Moreover, halides are among the key classes of compounds studied in thermodynamic contexts.

Compared with other halides, organic iodides are particularly important intermediates due to their ability to undergo nucleophilic substitution reactions under milder conditions than their lighter congeners. However, their preparation is somewhat more challenging than that of chlorides and bromides, as elemental iodine is less reactive than chlorine or bromine in direct halogenation reactions. Enhancing iodine’s electrophilic character typically requires polarization of the I_2_ molecule^1–3^ or, in more extreme cases, generation of an I^+^ species such as IPy_2_^+^ salts.^4–8^ Convenient iodinating agents include dichloroiodate (ICl_2_^–^) salts and iodine monochloride (ICl); this chemistry has been well-optimized and thoroughly documented.^9–23^

This work focuses on hypervalent iodine chemistry, which offers students strong theoretical foundations and valuable practical experience. Iodine stands out among the halogens for its ability to form a wide range of thermodynamically stable hypervalent compounds. These include inorganic species, such as iodonium salts, iodine oxides and oxoiodates, and halides/haloiodates, as well as organic compounds like diaryliodonium salts,^24,25^ acetoxy aryl iodine­(III) derivatives,^26^ iodosobenzene, iodoxybenzene, phenyliodine dichloride, and *o*-iodoxybenzoic acid and its triacetoxy derivative (the Dess–Martin periodinane).^27,28^ This molecular diversity offers valuable opportunities to deepen students’ understanding of both the VSEPR model and MO theory. The proposed experiment also introduces students to electrophilic iodination, particularly in the context of arene chemistry, from both theoretical and practical perspectives.

This experiment can be incorporated in the junior/senior-level laboratory course, either standing alone or linked to an Inorganic Chemistry lecture course. The prerequisite courses are two semesters of organic chemistry lecture and lab and one semester of inorganic or physical chemistry lecture; analytical chemistry lecture and lab are also welcomed. At the authors’ institution, this experiment is incorporated in the chemistry major capstone course called Synthesis. This material, along with its educational value, can also be useful to the practical benchtop chemists working in the research and development sector.

## Lecture

The introductory lecture typically begins with foundational inorganic chemistry of hypervalent halogens and concludes with an overview of the applications of selected halogen compounds in organic synthesis (lecture slides can be seen in the SI).

The most familiar halogen-containing compounds feature halogens in the univalent oxidation state, obeying the octet rule. Examples include halogen molecules X_2_, halide ions (X^–^), element halides with terminal halogen atoms, and organic halides, well-known representatives of both ionic and covalent species. There also exists a class of halides, such as Al_2_Cl_6_, in which some halogen atoms engage in extended valence bonding by donating lone pairs to form bridges between atoms. These bridging halogens still obey the octet rule due to the heterolytic nature of the additional bonding. In this lecture, we focus on another class of halogen compounds that is different from halides, namely, with halogen atoms in the valence state close to neutral or more often positive.

Following this theoretical foundation, the lecture presents the molecular orbital (MO) diagram of a diatomic halogen (X_2_), emphasizing how Lewis bases (B) weaken the X–X bond in the adducts with X_2_. This occurs because the only vacancy in the X_2_ molecule for this donor–acceptor interaction is in its antibonding σ_u_* orbital. In some cases, Lewis base adducts BXXB can be isolated; in other cases, complete cleavage of the X_2_ bond, usually heterolytic, as in reaction 1, is observed. This reactivity sets the stage for understanding halogen activation and the formation of electrophilic halogenating agents.

$$2I_{2}+2\mathrm{Py}⇆I{{\left(\mathrm{Py}\right)}_{2}}^{+}{I_{3}}^{-},\left(\mathrm{Py}=\mathrm{pyridine}\right)$$ 1

The linear geometry of the N–I–N and I–I–I units in the cation and anion shown above is explained using the VSEPR model; bonding in these ions is described using the MO model as 3c-2e, with the bond order of 1/2. The utility of the VSEPR model is further demonstrated using examples such as the ICl_4_^–^ ion, binary interhalogen compounds, and halogen oxoanions and acids.

The overview of hypervalent iodine compounds also includes organic (aryl) derivatives that serve as useful reagents: diaryliodonium salts^24,25^ used as arylating agents, phenyliodine dichloride used as a chlorinating agent, and acetoxy aryl iodine­(III) derivatives^26^ such as iodosobenzene, iodoxybenzene, *o*-iodoxybenzoic acid, and its triacetoxy derivative (the Dess-Martin periodinane),^27,28^ all used as mild and selective oxidizing agents.

The Lewis acidic behavior of interhalogen compounds is illustrated by reactions 2 and 3:

$$I_{2}{\mathrm{Cl}}_{6}+2{\mathrm{Cl}}^{-}\to 2{{\mathrm{ICl}}_{4}}^{-}$$ 2

ICl+Py→PyICl 3

The four compounds shown above, namely, ICl, I­(Py)_2_^+^, ICl_2_^–^ salts, and the PyICl adduct, all feature electrophilic iodine centers with strong potential for arene electrophilic substitution. The lecture concludes with a discussion of their applications in organic synthesis.

## Experiment

Many protocols for electrophilic iodination recommend using iodine monochloride ICl or dichloroiodate ICl_2_^–^ salts. The former can be prepared directly from elements,^29^ while the latter is accessible via either Lewis acid–base chemistry (ICl + Cl^–^ → ICl_2_^–^) or aqueous redox reactions (I^–^ + 2Cl^–^ → ICl_2_^–^ + 2e^–^).^12,30^ Dichloroiodate salts containing organic cations are appealing because they are nonvolatile and often air-stable, requiring no special handling, unlike ICl, which is volatile, corrosive, and toxic. A convenient alternative to ICl is the molecular adduct PyICl, formed from pyridine and ICl in nonaqueous solution.^31–33^

To prepare PyHICl_2_ and PyICl, we modified the reaction sequence to avoid the use of chlorine gas and iodine monochloride (ICl) as starting materials. Despite these changes, both target compounds were obtained in high yield. The new reaction sequence is described in the following paragraphs.

Comproportionation of iodate and iodide aided by low pH and complexation of iodine­(III) with chloride ligand yields potassium tetrachloroiodate (reaction 4).

$$2{\mathrm{KIO}}_{3}+\mathrm{KI}+12\mathrm{HCl}\to 3{\mathrm{KICl}}_{4}+6H_{2}O$$ 4

Tetrachloroiodic acid (HICl_4_), which is present in equilibrium with HCl in solution, exhibits strong affinity for ethers (reaction 5), facilitating its extraction. We used *tert*-butyl methyl ether (MTBE), which quantitatively extracts HICl_4_ (Figure 1). Adding pyridine to the MTBE solution of (R_2_OH)^+^ICl_4_^–^ (after drying with magnesium sulfate) causes the precipitation of pyridinium tetrachloroiodate. This salt can be optionally synthesized as an air-stable sparingly soluble yellow solid.

$${\mathrm{KICl}}_{4}+R_{2}O+\mathrm{HCl}\to {\left(R_{2}\mathrm{OH}\right)}^{+}{{\mathrm{ICl}}_{4}}^{-}+\mathrm{KCl},\left(R=\mathrm{alkyl}\right)$$ 5

**Figure 1:**
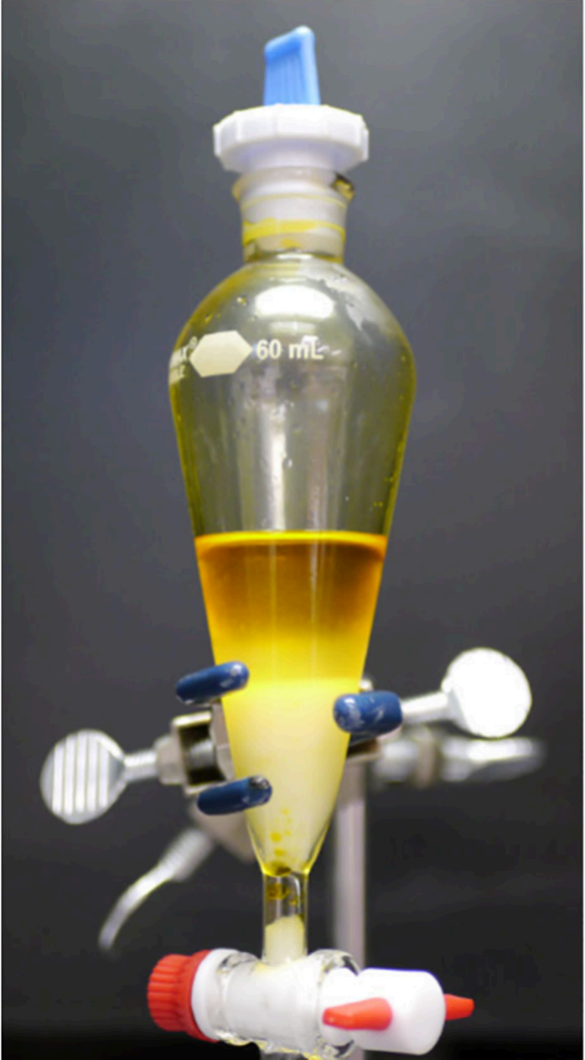
MTBE readily extracts HICl_4_ from the aqueous phase.

Under the suggested optimized conditions, acid-catalyzed hydrolysis of MTBE does not interfere with the desired reaction.

A comproportionation reaction between iodine­(III) and elemental iodine results in iodine­(I) compounds, dichloroiodic acid, and iodine monochloride (reaction 6).

$${\left(R_{2}\mathrm{OH}\right)}^{+}{{\mathrm{ICl}}_{4}}^{-}+I_{2}\to {\left(R_{2}\mathrm{OH}\right)}^{+}{{\mathrm{ICl}}_{2}}^{-}+2\mathrm{ICl}$$ 6

Pyridine reacts with dichloroiodic acid to form its pyridinium salt, while its reaction with iodine monochloride yields a covalent adduct (reaction 7). The target products PyHICl_2_ and PyICl precipitate out from solution and can be easily separated due to their different solubility properties.

$$\left(R_{2}\mathrm{OH}\right){\mathrm{ICl}}_{2}+2\mathrm{ICl}+3\mathrm{Py}\to {\mathrm{PyHICl}}_{2}+2\mathrm{PyICl}+R_{2}O,(\mathrm{Py}=\mathrm{pyridine})$$ 7

This reaction sequence offers several advantages: (a) all steps proceed under ambient conditions using basic glassware, (b) the stoichiometry of each reaction can be controlled and easily adjusted in the case of a deviation from the reaction protocol due to lack of accuracy, and (c) only products of reaction 7 are isolated from the solution. When the procedure is performed accurately, both reaction 7 products can be obtained as powdery solids in high yield. Separation is achieved using chloroform, which selectively dissolves the PyICl adduct. The crude product is isolated by rotary evaporation of the solution and then optionally purified by washing with absolute ethanol and/or MTBE, followed by drying under a nitrogen stream or vacuum. Both products are nonhygroscopic and can be handled on the benchtop in open air.

Since this method has not been previously reported, we performed analytical and spectrometric characterizations of both pyridinium dichloroiodate and the PyICl adduct. For comparison, PyICl was also synthesized using a literature method involving the reaction of ICl with pyridine in CCl_4_ solution (in course of this experiment development).^31^ Both samples were analyzed side by side and found to be identical (details are provided in the SI).

Salicylic acid was identified as a convenient substrate for electrophilic iodination. Both pyridinium dichloroiodate and the PyICl adduct are capable of affecting this reaction; however, we optimized the procedure for using the PyICl adduct. The crude adduct, isolated from a chloroform solution, is suitable for this purpose. The iodination reaction, carried out at ambient temperature in methanol, proceeds to completion within 1 h.

Depending on the chosen stoichiometry, the reaction yields either 3,5-diiodosalicylic acid or a mixture of the two monoiodosalicylic acid isomers. In this experiment, iodosalicylic acids were isolated free of pyridine and other organic impurities by extraction from a basic aqueous solution with MTBE, followed by reprecipitation using a mineral acid. The resulting iodosalicylic acids were essentially pure, as confirmed by both ^1^H and ^13^C NMR spectroscopy. Although the isomers could be separated chromatographically, this was not attempted; instead, the mixture was analyzed via the integration of diagnostic ^1^H NMR signals. Negative-mode electrospray (ESI) mass spectra and tandem (MS/MS) mass spectra were also acquired and interpreted.

## Spectrometric Characterization

The intermediate tetrachloroiodate, formed in reaction 4, was identified by negative-mode ESI mass spectrometry performed on a small sample of the reaction solution. The two most intense molecular ions, observed at *m*/*z* 267 (4 × ^35^Cl) and 269 (3 × Cl^35^ + ^37^Cl), exhibited the expected tandem MS fragmentation patterns, each showing a sequential loss of two Cl atoms, yielding *m*/*z* 232 and 197 and *m*/*z* 234 and 199, respectively.

For pyridinium dichloroiodate, the most intense mass spectral peak at *m*/*z* 197 showed a fragmentation pattern consistent with the expected product, with a major fragment at *m*/*z* 162 and a detectable ^35^Cl ion.

NMR characterization of pure PyHICl_2_ and PyICl was performed in deuterated acetone alongside free pyridine (Table 1 and Figure 2). The PyICl adduct synthesized by both methods displayed identical ^1^H NMR spectra.

**Table 1:** ^1^H NMR Data for Pyridine Ring Protons in Pyridine, PyHICl_2_, and PyICl in (CD_3_)_2_CO Solvent^a^

^1^H, ppm	Hydrogen in position 2	Hydrogen in position 4	Hydrogen in position 3
Pyridine	δ 8.576 (d, 4.1 Hz, 2H)	δ 7.750 (t, 7.6 Hz, 1H)	δ 7.340 (dd, 2H)
PyICl	δ 8.848 (d, 4.9 Hz, 2H)	δ 8.284 (t, 7.7 Hz, 1H)	δ 7.720 (m, 2H)
PyHICl_2_	δ 9.180 (d, 5.1 Hz, 2H)	δ 8.878 (t, 8.0 Hz, 1H)	δ 8.338 (m, 2H)

a Spectra were recorded on a Bruker AvanceCore 400 spectrometer.

**Figure 2:**
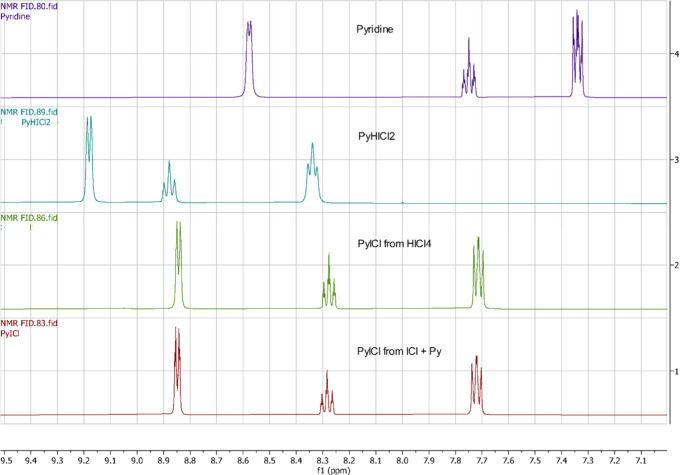
Stacked ^1^H NMR spectra of pyridine, PyHICl_2_, and PyICl made by two different methods. Spectra were recorded on a Bruker AvanceCore 400 spectrometer in (CD_3_)_2_CO solvent.

As can be seen, all three aromatic ^1^H NMR multiplets are shifted downfield in the bound pyridine, as compared to a free base; the triplet corresponding to the proton in the fourth position in PyH^+^ ion is most affected. The ^1^H and ^13^C NMR spectra of PyICl samples dissolved in CDCl_3_ are shown in the Supporting Information (Figures S1 and S2).

Iodosalicylic acids were identified by ESI mass spectrometry, with *m*/*z* = 263 and 389 corresponding to the mono- and disubstituted acids, respectively. Their tandem MS/MS spectra showed expected fragmentation patterns: for the monosubstituted acid, fragments at *m*/*z* 219 and 127; for the disubstituted acid, fragments at *m*/*z* 345, 217, and 127.

Proton NMR spectra of two products were analyzed. (Long-range H–H coupling was observed.) One product was prepared using a 1:2.25 molar ratio of salicylic acid to PyICl, and the other was prepared with a 1:1.1 ratio (Figures 3, S3, and S4). The spectra of the first product primarily contained 3,5-diiodosalicylic acid, with only 12 mol % of the 5-iodo isomer. The second one revealed a mixture of two monoiodosalicylic acid isomers (3-iodo and 5-iodo) and 3,5-diiodosalicylic acid in a molar ratio of 1.6:1:1 based on integration.

**Figure 3:**
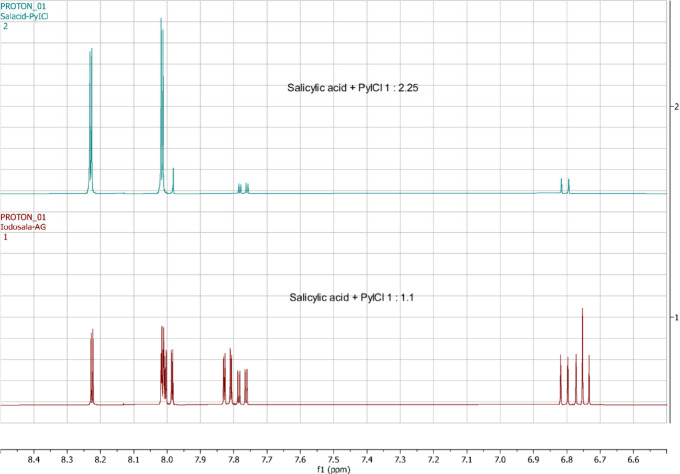
Stacked ^1^H NMR spectra of iodinated salicylic acids. Spectra were recorded on an Agilent 400MR spectrometer in (CD_3_)_2_SO solvent.

## Assessment and Evidence for Positive Learning Outcomes

Evaluation of student learning is conducted through multiple methods. A written lab report provides a detailed summary of the experimental results and discussion. A report guide is included in the lab manual and is available in the Supporting Information (SI). Submitted lab reports are carefully reviewed and graded.

To reinforce conceptual understanding, a study guide in the form of guiding questions is provided. Questions specific to halogen chemistry—including structure, bonding, and reactivity—assess both theoretical knowledge and practical application. These are incorporated into the quiz and the final exam. More general questions focused on spectral interpretation are used to evaluate students’ analytical skills and appear in problem sets as part of the final exam. An additional item being assessed is the *Substance-in-Use* data sheets (as seen in the next section).

Through these activities, students develop fluency and confidence in both their knowledge and their problem-solving abilities. For most students, scores on the spectrometry problem set included in the final exam are typically 28–35% higher than those on the comparable homework problem sets assigned earlier in the semester.

## Safety

The procedure is relatively safe and is carried out under (or close to) ambient conditions. The basic operations are familiar to junior- and senior-level students, and instruction on the use of a rotary evaporator is provided as needed. The reagent salts are safe for routine handling, and commonly used chemicals such as hydrochloric acid, methyl *tert*-butyl ether (MTBE), and chloroform are well-known to students from prior laboratory experience. For less commonly encountered reagents—namely elemental iodine and pyridine—each student completes a *Substance-in-Use* data sheet^34^ prior to the experiment. These sheets are reviewed and approved by the instructor and are discussed during the prelab instruction. According to SDSs available for 5-iodosalicylic and 3,5-diiodosalicylic acids at the Sigma-Aldrich Web site,^35^ both substances may produce a dust, which is an irritant for eyes and respiratory system. The instruction provided to students suggests handling dry powdery products under the fume hood (see the SI).

## Supplementary Material







## Notes

List of instruments used, analytical procedures and data, procedure details, study questions, lab report guidelines, and experiment timeline (PDF)
Hypervalent Halogens lecture slides (PDF)
NMR spectra for all compounds (PDF)

## References

[1] Hanson J. R. (2006). Advances
in the direct iodination of aromatic compounds. J. Chem. Res..

[2] Onto K., Hatakeyama T., Takeo M., Uchiito S., Tokuda M., Suginome H. (1997). Iodination
of Benzocyclic Amines With Mercury­(II) Oxide-Iodine
Reagent. Heterocyclic Commun..

[3] Stavber S., Kralj P., Zupan M. (2002). Progressive Direct Iodination of
Sterically Hindered Alkyl Substituted Benzenes. Synthesis.

[4] Hassel O., Hope H. (1961). Structure
of the solid compound formed by addition
of two iodine molecules to one molecule of pyridine. Acta Chem. Scand..

[5] Haque I., Wood J. L. (1968). The vibrational
spectra and structure of the bis­(pyridine)­iodine­(I),
bis­(pyridine)­bromine­(I), bis­(γ-picoline)­iodine-(I) and bis­(γ-picollne)­bromine­(I)
cations. J. Mol. Struct..

[6] Barluenga J., Rodriguez M. A., Campos P. J. (1990). Electrophilic Additions of Positive
Iodine to Alkynes through an Iodonium Mechanism. J. Org. Chem..

[7] Barluenga J., Gonzalez J. M., Garcia-Martin M. A., Campos P. J., Asensio G. (1992). An Expeditious
and General Aromatic lodination Procedure. J.
Chem. Soc., Chem. Commun..

[8] Barluenga J., Gonzalez J. M., Garcia-Martin M. A., Campos P. J., Asensio G. (1993). Acid-Mediated
Reaction of Bis­(pyridine)­iodonium­(I) Tetrafluoroborate with Aromatic
Compounds. A Selective and General Iodination Method. J. Org. Chem..

[9] Zefirov N.
S., Sereda G. A., Sosonuk S. E., Zyk N. V., Likhomanova T. I. (1995). Versatile
Iodination of Olefins by Potassium Dichloroiodate­(I). Synthesis.

[10] Garden S. J., Torres J. C., de Souza
Melo S. C., Lima A. S., Pinto A. C., Lima E. L. S. (2001). Aromatic
iodination in aqueous solution. A new lease
of life for aqueous potassium dichloroiodate. Tetrahedron Lett..

[11] Khalilzadeh M. A., Hosseini A. (2009). An easy, safe and simple
method for the iodination
of heterocyclic compounds in water. Iranian
J. Org. Chem..

[12] Larsen A. A., Moore C., Sprague J., Cloke B., Moss J., Hoppe J. O. (1956). Iodinated 3,5-Diaminobenzoic
Acid Derivatives. J. Am. Chem. Soc..

[13] Boyle M. (1909). The Iodobenzenemonosulfonic
Acids. Part I. J. Chem. Soc., Trans..

[14] Boyle M. (1911). Iodobenzenemonosulphonic
Acids. Part III. 2:3-diiodo- and 2:3:4:5-tetraiodobenzenesulfonic
Acids. J. Chem. Soc., Trans..

[15] Kajigaeshi S., Kakinami T., Moriwaki M., Tanaka T., Fujisaki S., Okamoto T. (1989). Halogenation Using
Quaternary Ammonium Polyhalides.
XIV. Aromatic Bromination and Iodination of Arenes by Use of Benzyltrimethylammonium
Polyhalides–Zinc Chloride System. Bull.
Chem. Soc. Jpn..

[16] Kajigaeshi S., Kakinami T., Watanabe F., Okamoto T. (1989). Halogenation using
quaternary ammonium polyhalides. XVII. Iodination of acetanilide with
benzyltrimethylammonium dichloroiodate and zinc chloride. Bull. Chem. Soc. Jpn..

[17] Filimonov V. D., Krasnokutskaya E. A., Lesina Yu. A. (2003). Generation of Electrophilic Iodine
from Iodine Monochloride in Neutral Media. Iodination and Protodeiodination
of Carbazole. Russ. J. Org. Chem..

[18] Krasnokutskaya E. A., Lesina Yu. A., Gorlushko D. A., Filimonov V. D. (2005). Comparative
Study of the Reactivity of Iodinating Agents in Solution and Solid
Phase. Russ. J. Org. Chem..

[19] Bahrami-Nasab S., Pourali A. R., Nazifi S. M. R. (2015). Direct
Iodination of Aromatic Compounds
by Using Polymer-Supported Dichloroiodate as an Efficient Reagent
under Mild Conditions. Natl. Acad. Sci. Lett..

[20] Vlassa M., Silberg I. A., Custelceanu R., Culea M. (1995). Reactions of π-Deficient
Aromatic Heterocycles with Ammonium Polyhalides I. Halogenation of
Acridone and Acridine Derivatives Using Benzyltriethylammonium (BTEA)
Polyhalides. Synth. Commun..

[21] Francke R., Schnakenburg G., Waldvogel S. R. (2010). Efficient and Reliable Iodination
and O-Methylation of Fluorinated Phenols. Eur.
J. Org. Chem..

[22] Hajipour A. R., Arbabian M., Ruoho A. E. (2002). Tetramethylammonium Dichloroiodate:
An Efficient and Environmentally Friendly Iodination Reagent for Iodination
of Aromatic Compounds under Mild and Solvent-Free Conditions. J. Org. Chem..

[23] Wallingford V. H., Krueger P. A. (1939). 5-Iodoanthranilic acid. Org.
Synth..

[24] Han J., Chen H., An G., Sun X., Li X., Liu Y., Zhao S., Wang L. (2021). Hypervalent
Iodonium Zwitterions
and Nucleophilic Aromatic Substitution: A Multiple-Step Experiment
in Organic Chemistry. J. Chem. Educ..

[25] Prendergast A. M., Shanahan R., Hickey A., Harrington F., Schönbauer D., Byrne P. A., Schnürch M., McGlacken G. P. (2020). Synthesis of a Diaryliodonium Salt and Its Use in the
Direct Arylation of Indole: A Two-Step Experiment for the Organic
Teaching Laboratory. J. Chem. Educ..

[26] Peng H.-C., Bryan J., Henson W., Zhdankin V. V., Gandhi K., David S. (2019). New, Milder Hypervalent
Iodine Oxidizing Agent: Using μ-Oxodi­(phenyliodanyl)
Diacetate, a (Diacetoxyiodo)­benzene Derivative, in the Synthesis of
Quinones. J. Chem. Educ..

[27] Dess D. B., Martin J. C. (1991). A Useful 12-I-5 Triacetoxyperiodinane
(the Dess-Martin
Periodinane) for the Selective Oxidation of Primary or Secondary Alcohols
and a Variety of Related 12-I-5 Species. J.
Am. Chem. Soc..

[28] Zhang J., Phillips J. A. (2010). The Synthesis and
Application of Polymer-Supported
Hypervalent Iodine Reagent in the Organic Chemistry Laboratory. J. Chem. Educ..

[29] Cornog, J. ; Karges, R. A. ; Test, L. A. Iodine Monochloride. In Inorganic Syntheses; McGraw-Hill Book Company, Inc., 1939; Vol. 1, p165.

[30] Gleu K., Jagemann W. (1936). Die Einwirkung von jodmonochloridlösung auf
heterocyclische basen. J. Prakt. Chem..

[31] Kauffman, G. B. ; Stevens, K. L. , Monopyridineiodine­(I) chloride. In Inorganic Syntheses; McGraw-Hill Book Company, Inc., 1963; Vol. VII, pp 176–180.

[32] Rømming C. H. R. (1972). Refinement of the Crystal
Structure of the Charge Transfer
Compound Pyridine-Iodomonochloride. Acta Chem.
Scand..

[33] Jones R. H., Knight K. S., Marshall W. G., Clews J., Darton R. J., Pyatt D., Coles S. J., Horton P. N. (2014). Colossal thermal
expansion and negative thermal expansion in simple halogen bonded
complexes. CrystEngComm..

[34] Kolesnichenko V. L., Goloverda G. Z. (2025). Substance-in-use data sheets for undergraduate synthesis
experiments. J. Chem. Educ..

[35] a 5-Iodosalicylic Acid (CAS RN: 119-30-2). I10600, Ver. 6.5. Sigma-Aldrich, September 6, 2024.

