# Supplementary material for: An Undergraduate Chemistry Experiment Integrating Theoretical and Practical Aspects of Hypervalent Iodine(I) Compounds
Source: J Chem Educ. 2026 Feb 27;103(3):1546–51. doi: 10.1021/acs.jchemed.5c01160 (PMC12980824; doi:10.1021/acs.jchemed.5c01160)
Supplement: Supplementary file 1 [file ed5c01160_si_001.pdf]

AN UNDERGRADUATE CHEMISTRY EXPERIMENT INTEGRATING THEORETICAL AND PRACTICAL ASPECTS OF HYPERVALENT IODINE(I) COMPOUNDS

Vladimir L. Kolesnichenko* and Galina Z. Goloverda*

E-mail vkolesni@xula.edu; gzgolove@xula.edu

Xavier University of Louisiana, Chemistry Department, 1 Drexel Dr., New Orleans, Louisiana 70125, United States

SUPPORTING INFORMATION

General All reagents and solvents were of the reagent grade purchased from ACROS or Aldrich. The solvents were pre-dried and stored over 3A molecular sieves. NMR spectra were recorded on Agilent 400MR, Bruker AvanceCore 400 and Anasazi Eft-90 spectrometers. Routine ESI mass-spectra were obtained on a Thermo Finnigan TSQ Ultra instrument. Gas chromatographic analysis was performed on Agilent 8860/5977 GC/MSD system.

Analysis

The $PyHICl_2$ samples were used as prepared; the PyICl adduct was washed with three portions of absolute ethanol (10, 5 and 5 mL per 2 g), followed by MTBE and dried in vacuum.

Iodine content was determined by digesting the 0.14-0.18 g sample in 25 mL of 0.1M NaOH (brief sonication helps to speed up), followed by addition of 10 mL of 0.6M KI, 3 mL of 1M H_2SO_4 and titrating with 0.07M standardized $Na_2S_2O_3$.

Pyridine content was determined by GC-MS method using an internal standard (acetophenone) for calibration. Samples (0.025-0.028 g) were combined with chloroform (9.0 mL) and digested by 50% aqueous NaOH (0.6 g). After vigorous stirring for 3 hours, a 5 mL aliquot of chloroform/pyridine solution was transferred into a 25-mL volumetric flask, combined with 2.0 mL of acetophenone standard solution (1.85 mg/mL) and diluted to 25 mL.

	PyICl (from ICl + Py)	PyICl (from $HICl_4$)	$PyHICl_2$ (from $HICl_4$)
Py found/calcd	32.33/32.73	32.63/32.73	28.75/28.44
I found/calcd	52.70/52.58	52.49/52.58	45.30/45.68

Procedure as provided in the lab manual (steps 1-4 are performed under a fume hood)

Reaction 1. Mix potassium iodate 2.14 g (10 mmol) with 0.83 g (5 mmol) of KI in a 100-mL Kjeldahl flask, add 1 mL of water, agitate and chill in ice/water slurry bath. Add 11 mL of concentrated (~12M) HCl by ~1 mL portions and agitate until the solids become homogeneous (5 minutes).

Reaction 2. Add 10 mL of pre-chilled in the ice bath MTBE directly into the reaction suspension and agitate until aqueous layer and the solid turn (almost) colorless, and the

MTBE layer turns intensely yellow (1 minute).[a] Transfer the liquids into a separatory funnel, allow layers to separate and drain the lower aqueous suspension into a receiver vessel (2 minutes). Transfer the MTBE solution into a dry 50 mL Erlenmeyer flask, rinse the separatory funnel with additional 1-2 mL of MTBE and combine this fluid with the main solution. Add the scoop of drying agent $MgSO_4$ and agitate well.[b] Separate the solution from solid drying agent by filtration, wash the solid with additional ~10 mL of MTBE and add this fluid to the main solution (5-10 minutes).

Reaction 3. Add the powderized iodine to the reaction solution in the amount of 3.80 g (15 mmol) according to stoichiometry of the reaction.[c] Stir the solution until iodine completely dissolves (25-30 minutes).[d]

Reaction 4. Combine 45 mmol of pyridine (3.56 g) with 20 mL of MTBE in a round-bottomed 100 mL flask with efficient spinbar.[e] Add the iodine chlorides solution to this solution dropwise while stirring (use an addition funnel). Slightly exothermic reaction results in massive precipitation of the yellow solid (5 minutes). At the end, stir the solution for additional ~30 minutes.

Workup. Filter the precipitate off and wash it with MTBE until the filtrate fluid turns almost colorless.[f] Discard the solution in a waste bottle. Separate the reaction products by selectively extracting on filter the soluble PyICl with ~80 mL of chloroform.[g] Dry the insoluble residue $PyHICl_2$ in a flow of nitrogen; weigh it to determine the yield. Transfer the filtrate solution into a pre-weighed 250-mL recovery flask and use rotary evaporator to evaporate the solvent and recover the solid PyICl product.[h] Weigh it to determine its yield. (~2 hours).[i]

Reaction 5. Dissolve 2 mmol of salicylic acid (276 mg) in 20 mL of methanol, add the powderized solid PyICl iodizer (2.2 or 4.5 mmol as suggested by instructor), cap the flask and stir the solution for 1 hour.

Neutralize the excess of PyICl by adding aqueous sodium metabisulfite "$Na_2S_2O_5$" (0.5 mmol, 95 mg) in ~1 mL of water. Remove the spinbar, transfer the solution into 100-mL roundbottom flask with 24/40 ground joint and evaporate the solvent using rotary evaporator. Wash the residue with ~20 mL of water, then dissolve it in 40 mL of 0.1M NaOH (pH should be basic). Extract pyridine with 2x(10 mL) of MTBE and discard the organic layer. Acidify the aqueous layer with 0.1M HCl (solution should be acidic), heat the milky suspension in boiling water bath for ~3 minutes, allow it to cool naturally. Filter the precipitate off on the pre-weighed fritted glass filter and wash it with water. (1-1.5 hours)

Hold the filter with the product over pre-weighed 50-mL beaker, add ~2 mL of acetone, cover the filter with aluminum foil and agitate gently. Push the solution down from the filter using a stopper-tube adapter and a rubber bulb. Add additional ~2 mL of acetone and repeat the above procedure. If the product dissolved incompletely, repeat the acetone wash step again. Leave the beaker with solution in the fume hood and allow the solution to air-dry.[j] Weigh the beaker with dry solid to determine the product yield. Obtain the ESI mass spectrum and NMR spectra in d-DMSO solvent.

Notes:

[a] Using more MTBE will extract HCl from aqueous phase and thus will affect the yield of the target products.

[b] If the whole amount of the drying agent clumps, add more drying agent and make sure a part of it remains powdery.

[c] Iodine must be ground in mortar.

[d] Spillage of chloroiodate and ICl solutions can be neutralized with aqueous sodium thiosulfate.

[e] Precipitating reaction product makes the suspension thick which might affect the efficiency of stirring and cause the undesired inhomogeneity.

[f] Use a pre-weighed 60-mL fritted glass filter. Preferably, apply the pressure from top instead of suctioning. For washing, use ~60 mL of MTBE.

[g] A small amount of $PyHICl_2$ may crystallize in the filtration flask, which can be separated by decanting.

[h] The optimal rotavapping conditions are: water bath temperature 60°C, ice-chilled condenser and vacuum 350-360 torr.

[i] The product can be optionally washed with ~30 mL of absolute ethanol and/or MTBE and dried in a nitrogen flow or in vacuum.

[j] Dry solid easily forms the dust which irritates eyes and respiratory system, so it should be handled under the fume hood.

Characterization

- Take a minute-sized sample of $HICl_4$ solution and examine it using an electrospray (ESI) mass-spectra in a negative ms1 and fragmentation (ms2) modes. Interpret these spectra.
- Take a minute-sized sample of iodosalicylic acid you have synthesized and examine it using an electrospray (ESI) mass-spectra in a negative and fragmentation modes. Interpret these spectra.
- Take and interpret ^1H and ^{13}C NMR spectra of both $PyHICl_2$ and PyICl in CD_3CN solvent.
- Take and interpret ^1H and ^{13}C NMR spectra of iodosalicylic acid in d-DMSO solvent.

Study questions (use the "Hypervalent Halogens" handout)

- What happens to halogen molecules interacting with Lewis bases? What are the extreme cases? What actually causes this change?
- What is the molecular structure of $I(Py)_2^+$ cation, ICl_2^- anion and PyICl adduct (consider only N atom in pyridine)? Rationalize it in terms of VSEPR model.
- What is the molecular structure of tetrachloroiodate ion? Rationalize it in terms of VSEPR model.
- What is the bond order in dichloroiodate ion based on MO model?

- Write the balanced equations of the formation of iodine monochloride and iodine trichloride.
- Write the balanced equations for two different methods of the formation of dichloroiodate ion.
- Write a balanced equation of the reaction between pyridine and dichloroiodic acid.
- Write a balanced equation of the reaction between pyridine and iodine monochloride.
- Sketch a reaction scheme showing PyICl acting as electrophilic iodinating agent on benzene derivatives.

Lab Report Instructions

The objectives of this experiment listed in the introduction: getting familiar with synthesis, structure and reactivity of iodine chloride compounds. Exploring their application as electrophilic iodinating agents for benzene derivatives. Learning the NMR and mass-spectrometry interpretation strategies.

In the Discussion section, outline the reactions of formation of each intermediate and target product. Discuss the properties of each intermediate and product. It is okay to use the reaction schemes from the provided handout. Add the relevant schemes absent in it.

Comment on the spectrometric identification of all products. In ^{1}H NMR, identify each proton signal, its position, multiplicity (coupling) and integral. In ^{13}C spectra, identify each carbon signal and state if the numbers match. Comment on NMR purity of all products.

Look at the NMR spectra of different compounds side-by-side and track what happens to this or that proton or carbon as the molecule is modified.

In the Experimental section, describe your observations after each step of synthesis and associate each observation with the chemical reaction occurred. Check the provided guide for other items in the contents of this section.

For the Conclusions section, comment on what you have learned; also refer to items listing in the provided guide.

Timeline

Day 1. Lecture (60-80 minutes). Experimental techniques instruction. Safety comments, including Data Sheets discussion (20 minutes). Glassware, equipment setup; chemicals measuring out (30 minutes). Reaction 1 (5 minutes). Reaction 2 (10 minutes). Reaction 3 (30 minutes). Reaction 4 (30 minutes).

Day 2. Solid products filtration, washing and separation using extraction with chloroform. Recovery of two products: drying in a flow of nitrogen; solvent evaporation using a rotary evaporator (2-2.5 hours). Reaction 5 (70 minutes): as soon as this reaction is set up, students carry out spectrometric characterization of PyICl and $PyHICl_2$ products, which can take an additional 0.5-1 hour. Reaction 5 quenching (5 minutes).

Day 3. Reaction 5 workup and product recovery (1.5 hours). Iodosalicylic acids' spectrometric characterization (1.5 hours). Final discussion and lab report guidelines (1 hour).
