# Supplementary material for: An Undergraduate Chemistry Experiment Integrating Theoretical and Practical Aspects of Hypervalent Iodine(I) Compounds
Source: J Chem Educ. 2026 Feb 27;103(3):1546–51. doi: 10.1021/acs.jchemed.5c01160 (PMC12980824; doi:10.1021/acs.jchemed.5c01160)

An Undergraduate Chemistry Experiment Integrating Theoretical and Practical Aspects of Hypervalent Iodine(I) Compounds

Vladimir L. Kolesnichenko * and Galina Z. Goloverda *

Xavier University of Louisiana, Chemistry Department, 1 Drexel Dr., New Orleans, Louisiana 70125, United States

Supporting Information: Lecture slides

HALOGENS

	1	2	3	*	4	5	6	7	8	9	10	11	12	13	14	15	16	17	18
1	H 1	He 2																H 1	He 2
2	Li 3	Be 4												B 5	C 6	N 7	O 8	F 9	Ne 10
3	Na 11	Mg 12												Al 13	Si 14	P 15	S 16	Cl 17	Ar 18
4	K 19	Ca 20	Sc 21		Ti 22	V 23	Cr 24	Mn 25	Fe 26	Co 27	Ni 28	Cu 29	Zn 30	Ga 31	Ge 32	As 33	Se 34	Br 35	Kr 36
5	Rb 37	Sr 38	Y 39		Zr 40	Nb 41	Mo 42	Tc 43	Ru 44	Rh 45	Pd 46	Ag 47	Cd 48	In 49	Sn 50	Sb 51	Te 52	I 53	Xe 54
6	Cs 55	Ba 56	La 57	* 58-71	Hf 72	Ta 73	W 74	Re 75	Os 76	Ir 77	Pt 78	Au 79	Hg 80	Tl 81	Pb 82	Bi 83	Po 84	At 85	Rn 86
7	Fr 87	Ra 88	Ac 89	** 90-103	104	105	106	107	108	109	110	111	112	113	114	115	116	117	118

*	Ce 58	Pr 59	Nd 60	Pm 61	Sm 62	Eu 63	Gd 64	Tb 65	Dy 66	Ho 67	Er 68	Tm 69	Yb 70	Lu 71
**	Th 90	Pa 91	U 92	Np 93	Pu 94	Am 95	Cm 96	Bk 97	Cf 98	Es 99	Fm 100	Md 101	No 102	Lr 103

COMPOUNDS OF HYPERVALENT HALOGENS

The most familiar type of halogen compounds contain halogen in a univalent state obeying the octet rule. Halogen molecules X_2, halide ions X-, element halides with terminal X atoms, and organic halides are best-known examples of such ionic or covalent species.

There is also a group of halides such as Al_2Cl_6 in which some halide atoms extend their valent bonding to two or more by utilizing one or more of their lone pairs, and thus act as bridging. These halogen atoms still obey the octet rule due to heterolytic nature of their “additional” bonding.

This chapter introduces a class of compounds that is different from halides, namely with halogen atoms in their valent state close to neutral or more often positive.

Molecular Orbitals (MO) diagram of a halogen (X_2) molecule

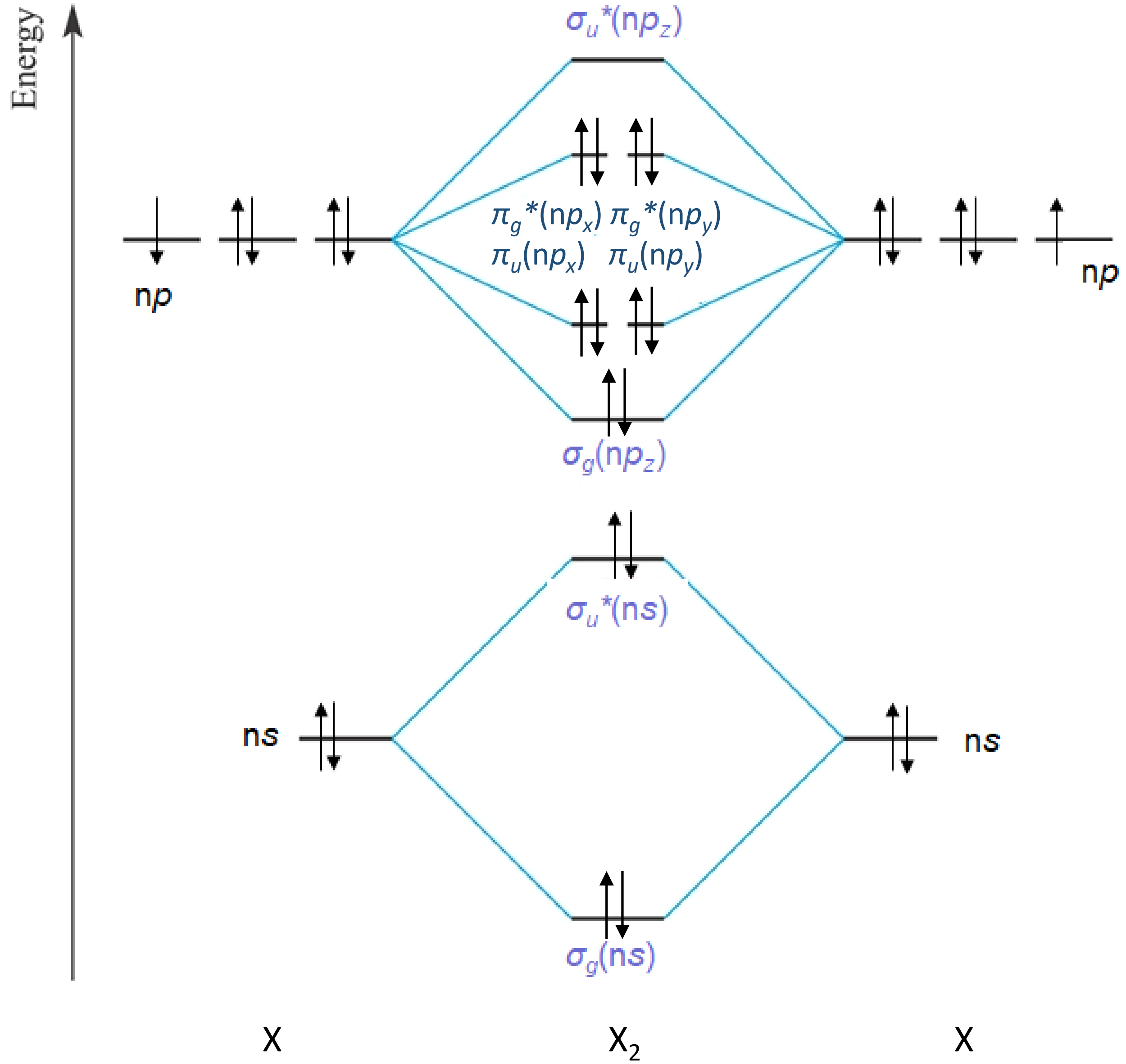

Bond order = (# of bonding electrons - # of antibonding electrons)/2 = (8-6)/2 = 1

Molecular Orbitals (MO) diagram of X_2 molecule

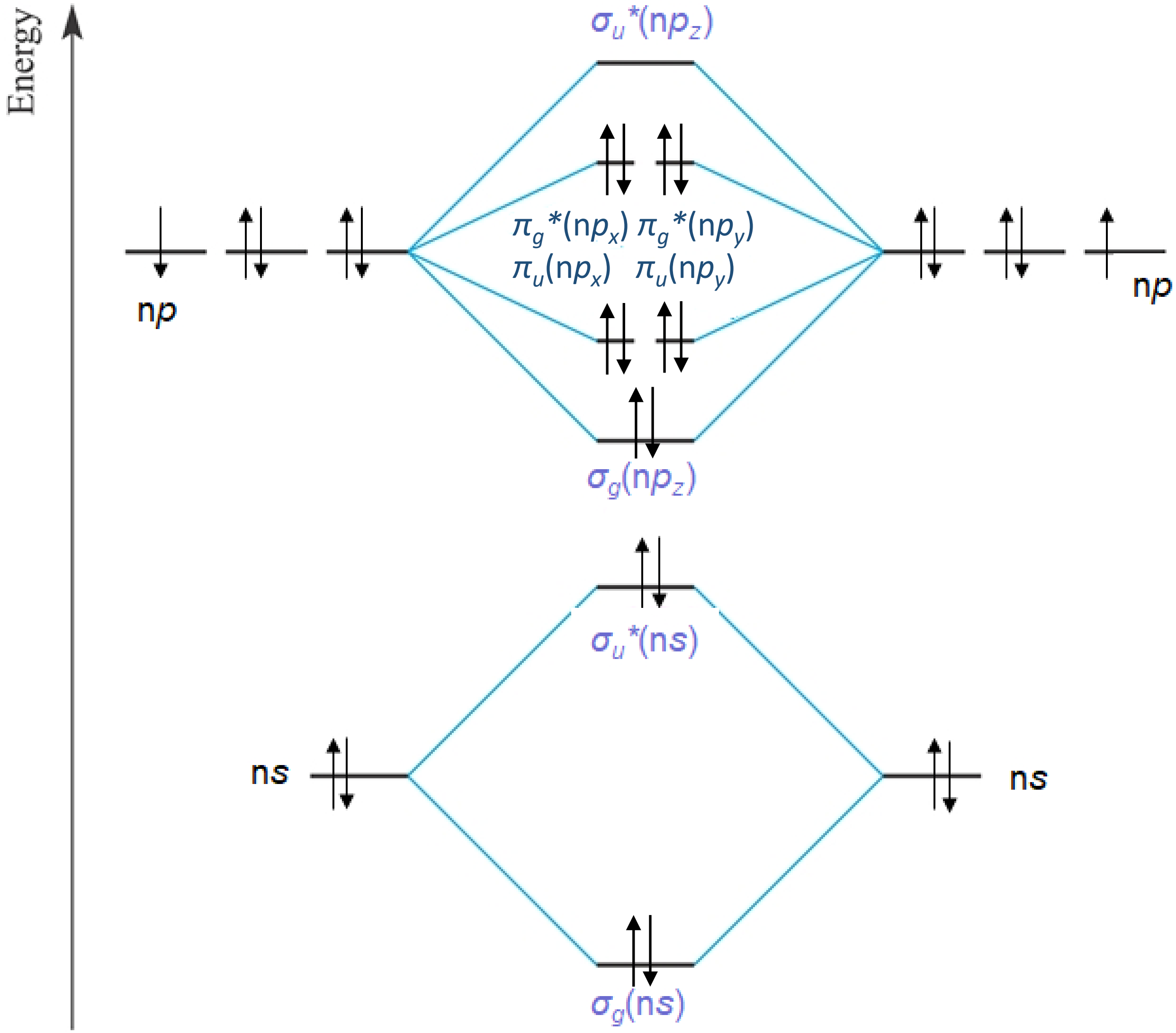

Adding electrons to the Lowest Unoccupied Molecular Orbital (LUMO), which is antibonding, reduces the bond order and thus destabilizes the molecule.

Adding electrons to LUMO, which is antibonding, reduces the bond order and thus destabilizes the molecule

In the extreme, the X_2 molecule breaks apart upon two-electron reduction: $X_2 + 2e^- \rightarrow 2X^-$

Lewis bases do it more elegantly: the molecule might survive, but the X-X bond weakens:

B:→X-X   Br-Br bond length trends: 228 pm in Br_2,
233 pm in $CH_3CN \cdots Br\text{-}Br \cdots NCCH_3$,
241 pm in ArEtS···Br-Br···SEtAr
312 pm in $Ph_3P \cdots Br\text{-}Br$

In another extreme, the X_2 molecule breaks apart in reactions with a very strong base:

$$X_2 + 2OH^- \rightarrow X^- + OX^- + H_2O$$

This is a disproportionation (self-oxidation-reduction) reaction where the X-X bond breaks heterolytically: X:X → X: + X or in a different notation X-X → X^- + X^+

Selection and bond length in bromine adducts were taken from C. Housecroft and A.G. Sharpe, *Inorganic Chemistry*, 5th Edition, Pearson 2018

Special case of disproportionation - iodine

$2I_2 + 2Py \leftrightarrows I(Py)_2^+I_3^-$

$I_2 + AgClO_4 + 2Py \longrightarrow I(Py)_2^+ClO_4^- + AgI$

Py = pyridine

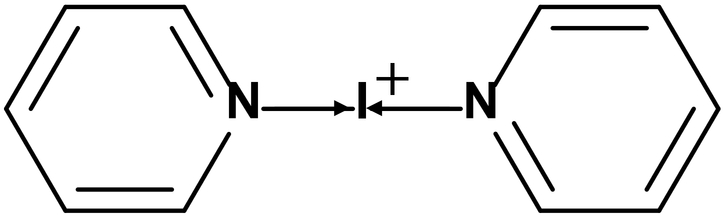

Polyhalide ions are best represented by Br_3^- and I_3^-

Tribromide ion is well-known as a part of crystalline solids, but dissociates in aqueous solutions: $Br_2 + Br^- \leftrightarrows Br_3^-$

Triiodide ion is more stable as it is well-known in crystalline solids and in aqueous solutions: $I_2 + I^- \leftrightarrows I_3^-$

Both ions have a linear structure X—X—X with equal bonds around central atom.*
This structure can be described using VSEPR model: the orbital symmetry is trigonal bipyramidal with non-bonding orbitals occupying equatorial positions:

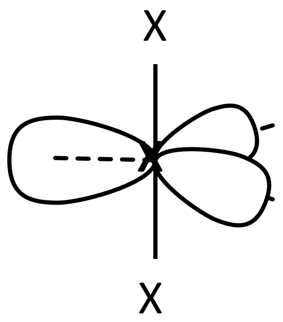

* Bond length is a subject to variation in crystalline solids

Interhalogen polyhalide ions

Similarly to the reaction $I_2 + I^- \leftrightarrows I_3^-$, iodine element can be replaced by chlorine: $Cl_2 + I^- \rightarrow ICl_2^-$. Iodine has the oxidation number of +1 in this dichloroiodate ion. It shares the same molecular structure with its triiodide congener:

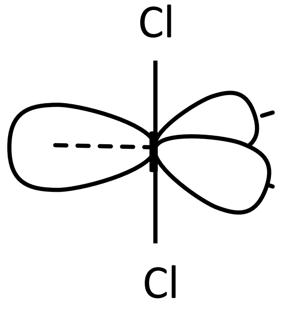

Further chlorination results in the formation of tetrachloroiodate ion: $Cl_2 + ICl_2^- \rightarrow ICl_4^-$. Iodine has the oxidation number of +3 and its structure can also be described using VSEPR model: the orbital symmetry is octahedral with two non-bonding orbitals occupying axial positions. Four bonding orbitals are equal and positioned in the square plane.

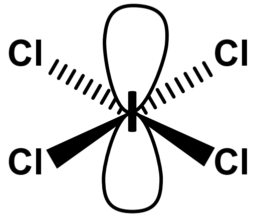

Molecular Orbitals (MO) diagram of ICl_2^- ion derived from p_z atomic orbitals

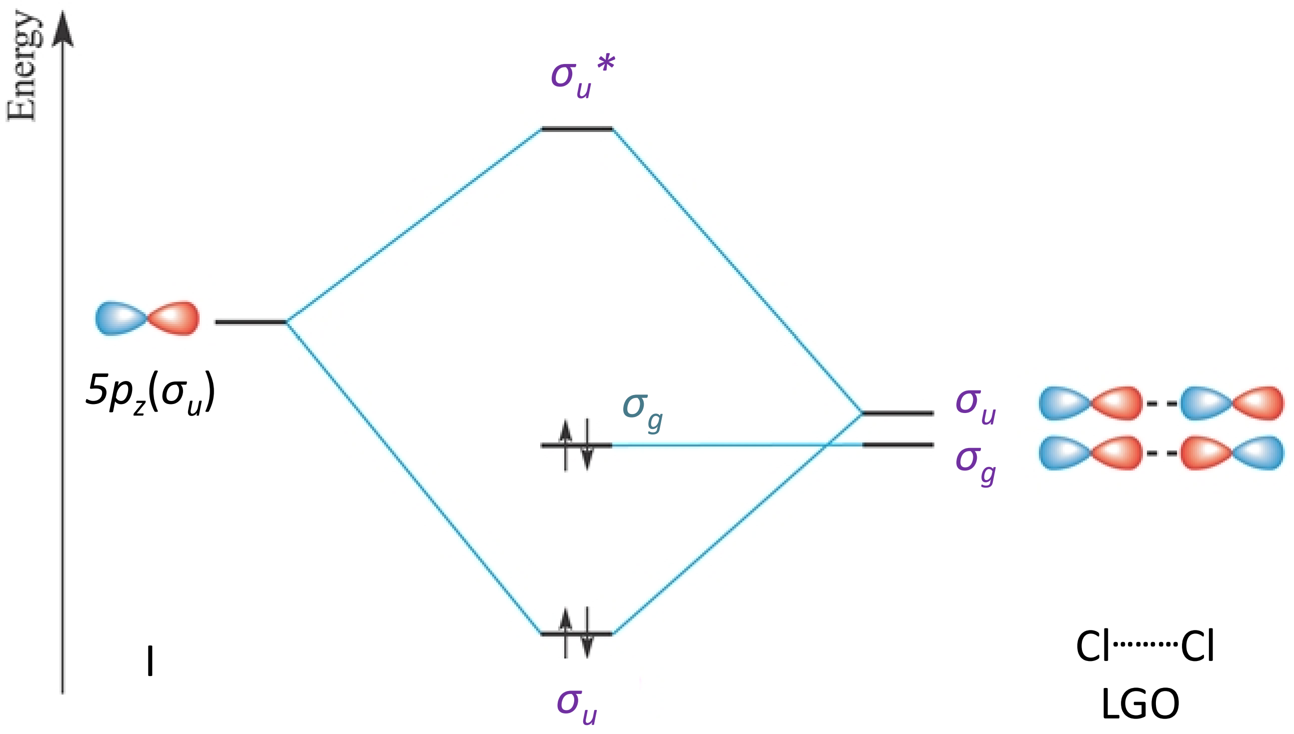

Note: there is only one occupied bonding MO (σ_u) per two I-Cl bonds making the bond order ½.

Similar diagram might be applied to σ-bonding in $I(Py)_2^+$ ion, however π-bonding can possibly be involved too.

Binary interhalogen compounds

Similarly to the reaction $Cl_2 + I^- \rightarrow ICl_2^-$, iodide ion can be replaced with iodine element: $Cl_2 + I_2 \rightarrow 2ICl$. The product is iodine monochloride whose molecule is similar to Cl_2 or I_2, but polar.

Similarly to the reaction $Cl_2 + ICl_2^- \rightarrow ICl_4^-$, dichloroiodate ion can be replaced with iodine monochloride: $Cl_2 + ICl \rightarrow ICl_3$. The product is iodine trichloride whose molecule is a planar dimer made of two fused squares:

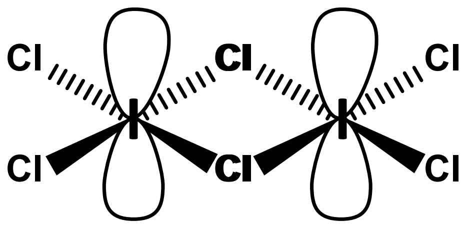

This structure can be understood as a result of Lewis acid-base interactions with iodine in ICl_3 acting as a Lewis acid and chlorine from another ICl_3 as a Lewis base:

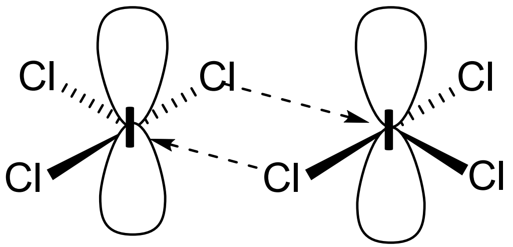

Iodine chlorides as Lewis acids

Both iodine chlorides act as Lewis acids in reaction with chloride ion:

$ICl + Cl^- \longrightarrow ICl_2^-$ $\qquad$ $I_2Cl_6 + 2Cl^- \longrightarrow 2ICl_4^-$

Both reactions offer alternative paths to the synthesis of dichloroiodate and tetrachloroiodate salts, as compared to direct chlorination of iodide shown above.

Iodine monochloride is also known to react with pyridine (a Lewis base) to produce a molecular adduct $ICl + Py \longrightarrow PyICl$ which structurally relates to both $I(Py)_2^+$ and ICl_2^-.

Oxidation-reduction properties of iodine chlorides

Like all halogens, iodine chlorides are aggressive oxidizing agents. They are volatile, corrosive to many materials and toxic acting on respiratory system and skin. They can chlorinate many elements:

$$2Fe + 6ICl \rightarrow 2FeCl_3 + 3I_2$$

$$2Au + 3ICl_3 \rightarrow 2AuCl_3 + 3ICl$$

Chloroiodates are also strong oxidizing agents, but they are much friendlier for handling because they are not volatile.

Interhalogen compounds
Synthesis

Most common synthesis methods involve elements:

$Cl_2 + F_2 \leftrightarrows 2ClF$ colorless gas

$Cl_2 + 3F_2 \rightarrow 2ClF_3$ colorless gas

$Cl_2 + 5F_2 \rightarrow 2ClF_5$ colorless gas

$Br_2 + F_2 \rightarrow 2BrF$ yellow gas

$Br_2 + 3F_2 \rightarrow 2BrF_3$ yellow liquid

$Br_2 + 5F_2 \rightarrow 2BrF_5$ colorless liquid

$Br_2 + Cl_2 \leftrightarrows 2BrCl$ yellow gas

$I_2 + 5F_2 \rightarrow 2IF_5$ colorless liquid

$I_2 + 7F_2 \rightarrow 2IF_7$ colorless gas

$I_2 + Cl_2 \rightarrow 2ICl$ dark-red solid

$I_2 + 3Cl_2 \rightarrow I_2Cl_6$ deep-orange solid

$I_2 + Br_2 \rightarrow 2IBr$ dark-red solid

Halogen Fluorides and Fluoro complexes

Valence orbital geometry

Oxidation number	+1	+3	+5	+7
Cl	ClF 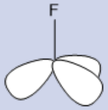	ClF_3 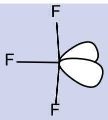	ClF_5 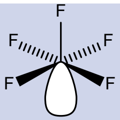	
Cl		ClF_4^- 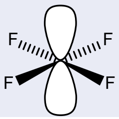	ClF_6^- Non-rigid octahedron	
Br	BrF 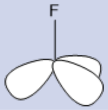	BrF_3 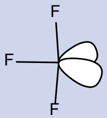	BrF_5 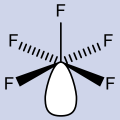	
Br		BrF_4^- 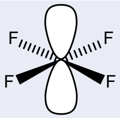	BrF_6^- Non-rigid octahedron	
I			IF_5 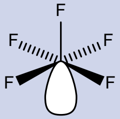	IF_7 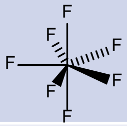
I			IF_6^- Non-rigid octahedron	IF_8^- Square antiprism
I				IF_6^+ 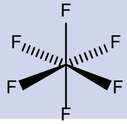

Halogen Chlorides and Chloro complexes

Valence orbital geometry

Oxidation number	+1	+3	+5	+7
Br	BrCl 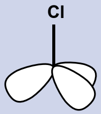			
I	ICl 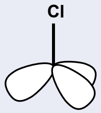	I_2Cl_6 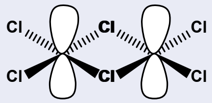		
I	ICl_2^- 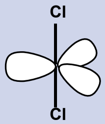	ICl_4^- 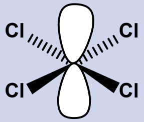		
I		ICl_2^+ 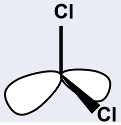		

Valence orbital geometry of halogen oxoanions and oxoacids

Halogen oxidation number (X = Cl, Br, I)

+1	+3	+5	+7
Hypohalites<br>XO^- 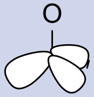	Chlorite<br>ClO_2^- 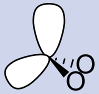	Halates<br>XO_3^- 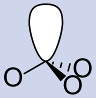	Perhalates<br>XO_4^- 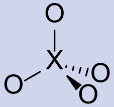
Hypohalous acids<br>HOCl<br>HOBr<br>HOI	Chlorous acid<br>$HClO_2$	Halic acids<br>$HClO_3$<br>$HBrO_3$<br>HIO_3	Perhalic acids<br>$HClO_4$<br>$HBrO_4$<br>HIO_4, H_5IO_6

The extensive chemistry of halogen oxoacids is described in Chap. 17

Organic compounds with hypervalent iodine

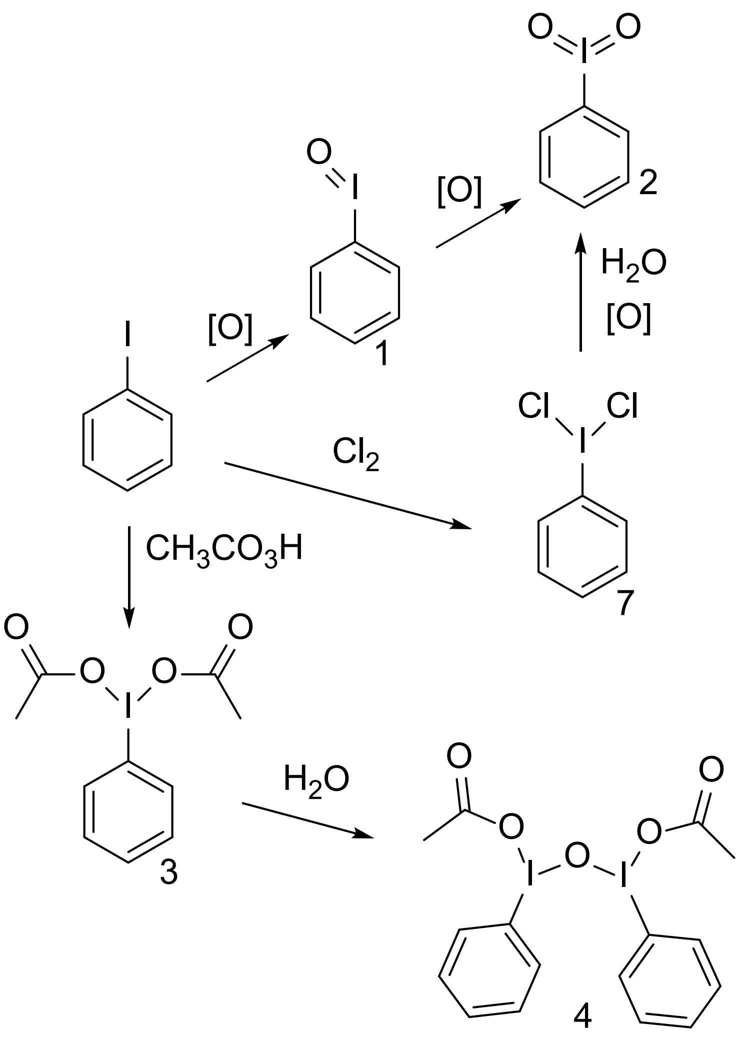

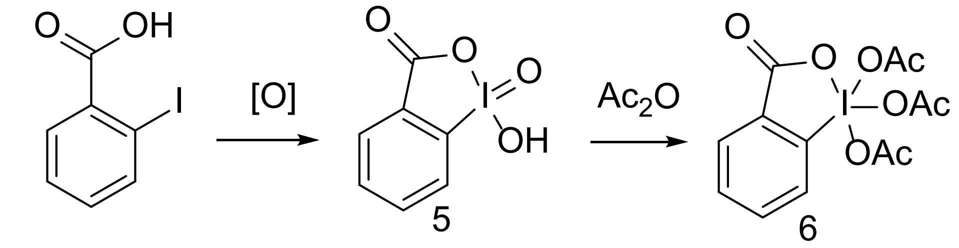

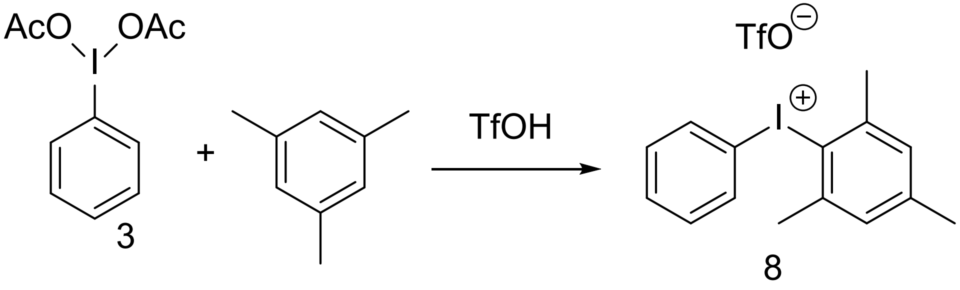

1-6 – Oxidizing agents

7- Chlorinating agent

8 – arylating agent

In this experiment

A convenient synthesis method proposed here (avoiding using chlorine or iodine chlorides) is based on comproportionation of iodate and iodide aided by low pH and complexation of positive-valent iodine with chloride ligand (reaction 1).

1. $2KIO_3 + KI + 12HCl \rightarrow 3KICl_4 + 6H_2O$
2. $KICl_4 + R_2O + HCl \rightarrow (R_2OH)ICl_4 + KCl$
3. $(R_2OH)ICl_4 + I_2 \rightarrow (R_2OH)ICl_2 + 2ICl$
4. $(R_2OH)ICl_2 + 2ICl + 3Py \rightarrow PyHICl_2 + 2PyICl + R_2O$ Py = pyridine

Tetrachloroiodic acid $HICl_4$ present in the equilibrium hydrochloric acid solution, exhibits a strong affinity to ethers (reaction 2) which aids its extraction.

The redox reaction between iodine(III) and iodine element results in iodine(I) compounds, dichloroiodic acid and iodine monochloride (Reaction 3).

Pyridine reacts with dichloroiodic acid yielding its pyridinium salt; pyridine reacting with iodine monochloride yields a covalent adduct (reaction 4). The target products $PyHICl_2$ and PyICl are easily separated thanks their different solubility properties.

Both compounds are tested for the electrophilic aromatic iodination; the reaction in line notation is ArH + PyICl → ArI + PyHCl

Electrophilic Aromatic Substitution Reaction (EAS)

- The most characteristic reaction of aromatic hydrocarbons (arenes) is their reaction with electrophilic reagents which results in substitution of hydrogen for the electrophile at the aromatic ring.

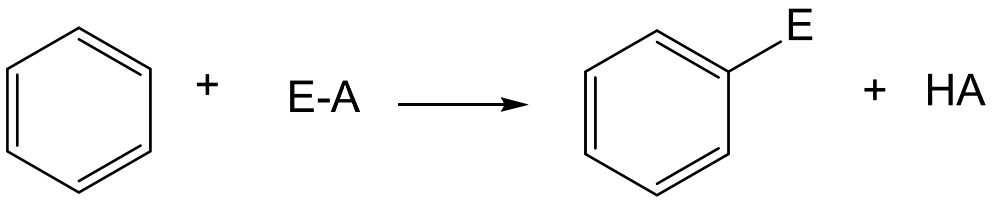

E - electrophile

In more general form:

$$\text{Ar-H} + \text{E-A} \longrightarrow \text{Ar-E} + \text{HA}$$

- In case of electrophilic iodination "I^+" is the actual electrophile.

EAS iodination mechanism of salicylic acid

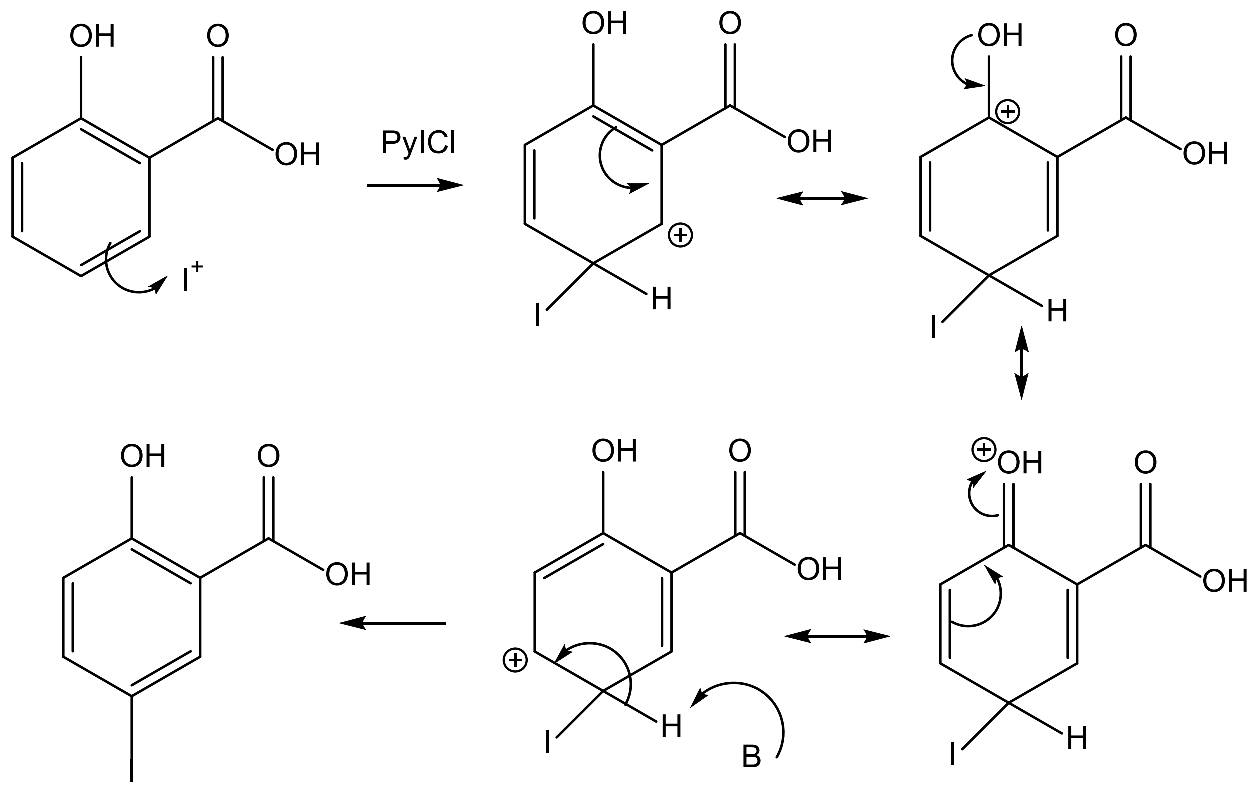

Supplement: Supplementary file 2 [file ed5c01160_si_002.pdf]
