# Supplementary material for: An Undergraduate Chemistry Experiment Integrating Theoretical and Practical Aspects of Hypervalent Iodine(I) Compounds
Source: J Chem Educ. 2026 Feb 27;103(3):1546–51. doi: 10.1021/acs.jchemed.5c01160 (PMC12980824; doi:10.1021/acs.jchemed.5c01160)
Supplement: Supplementary file 3 [file ed5c01160_si_003.pdf]

An Undergraduate Chemistry Experiment Integrating Theoretical and Practical Aspects of Hypervalent Iodine(I) Compounds

Vladimir L. Kolesnichenko * and Galina Z. Goloverda *

Xavier University of Louisiana, Chemistry Department, 1 Drexel Dr., New Orleans, Louisiana 70125, United States

Supporting Information: NMR spectra

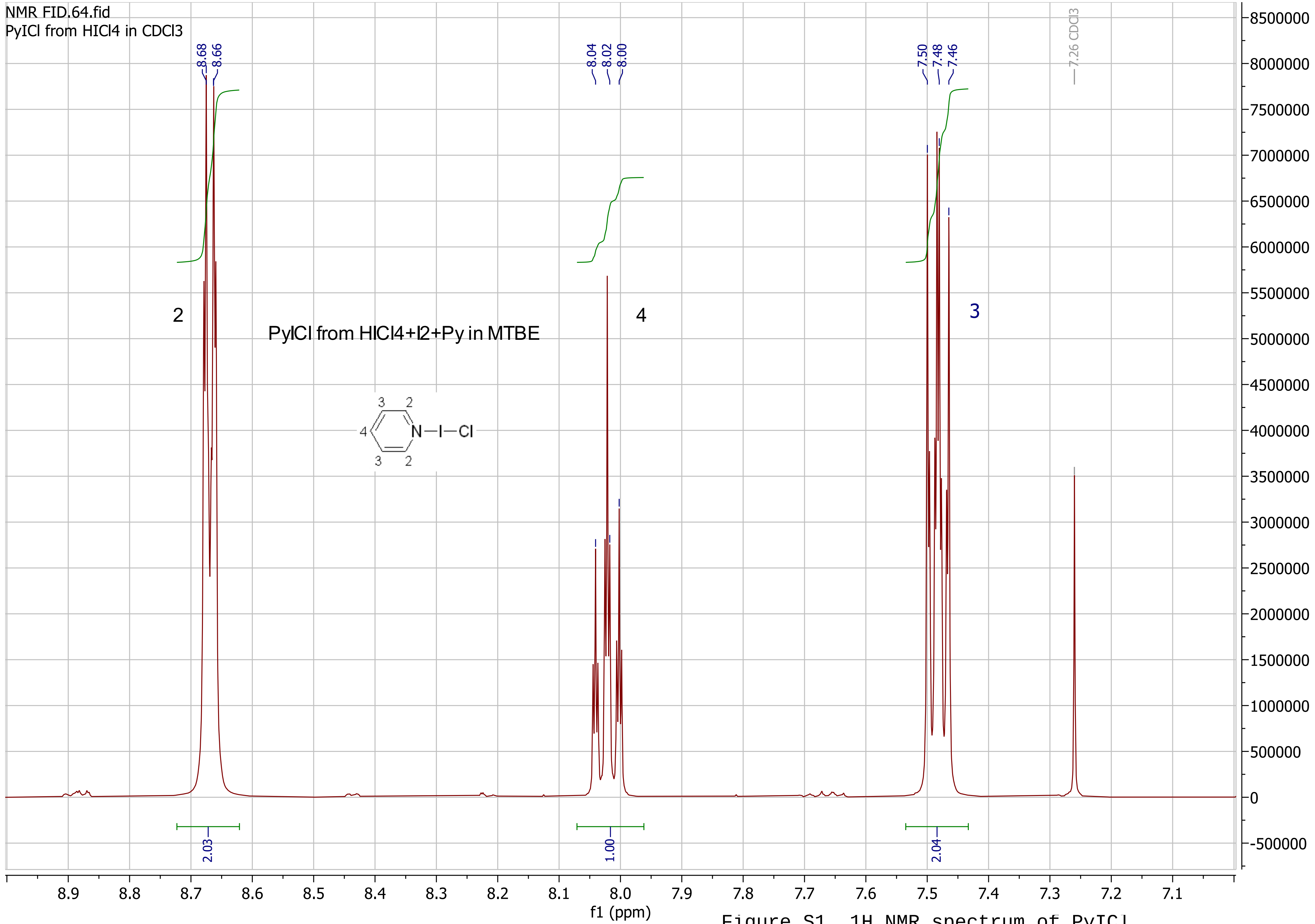

Figure S1. 1H NMR spectrum of PyICl

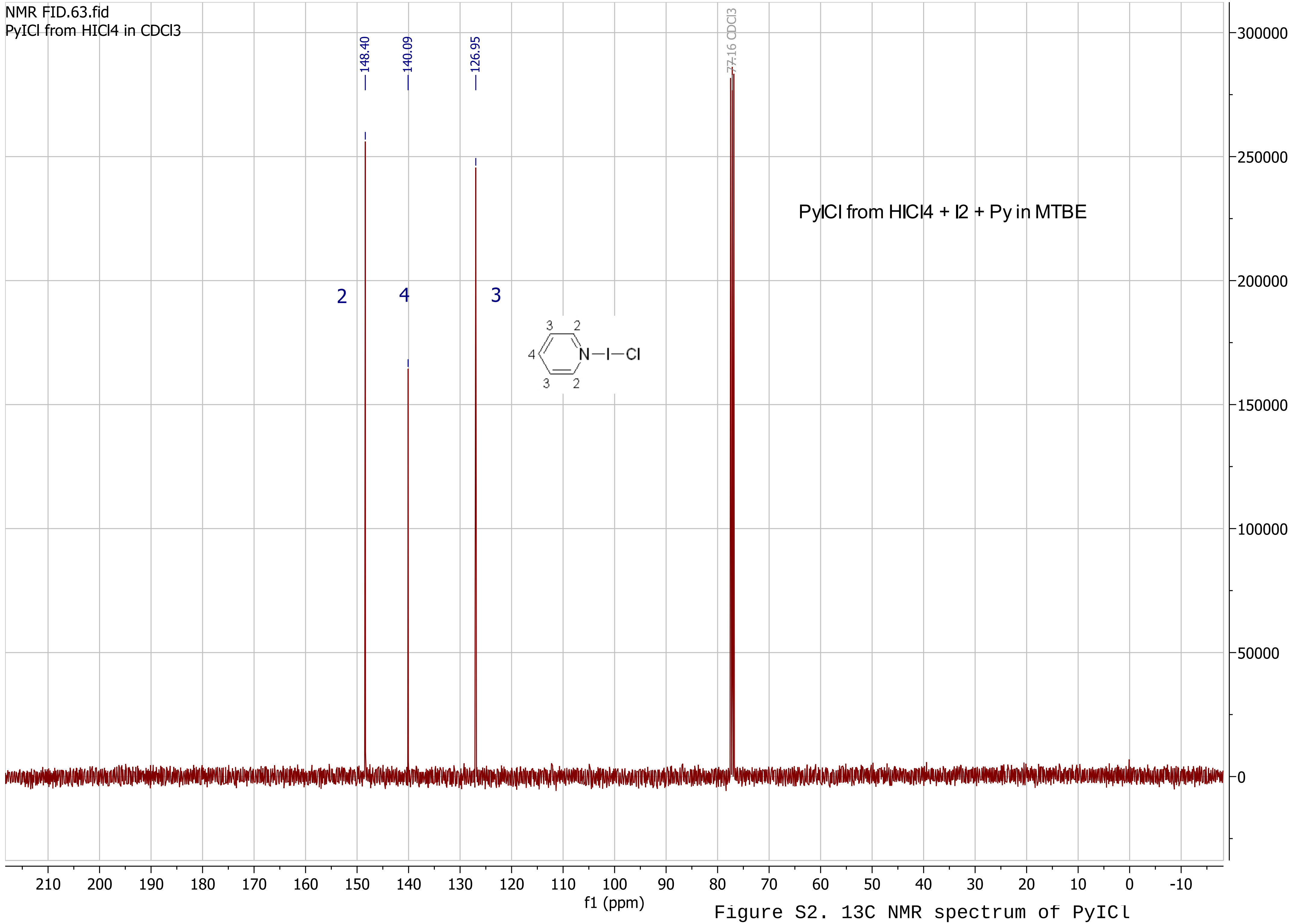

Figure S2. 13C NMR spectrum of PyICl

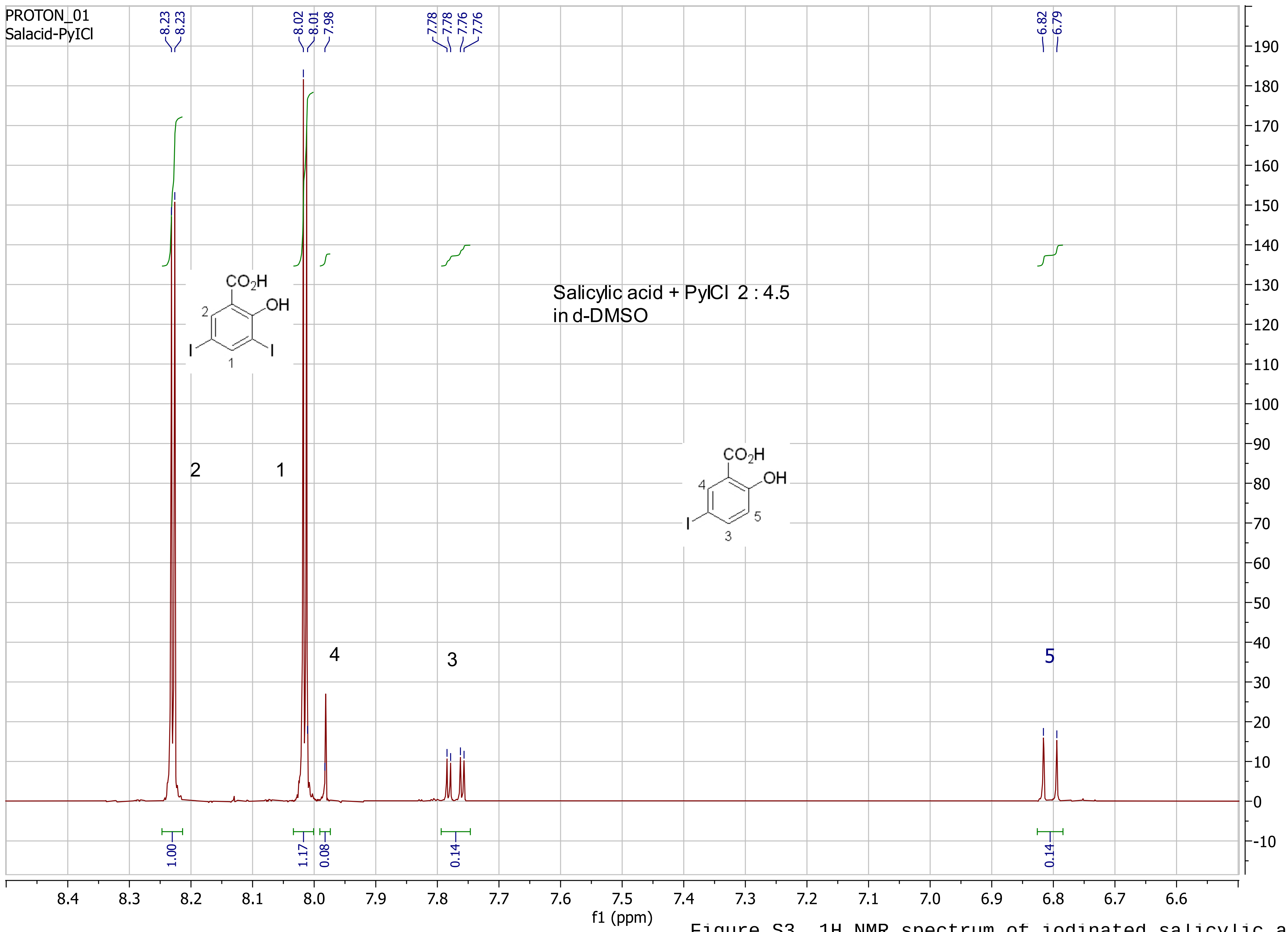

Figure S3. 1H NMR spectrum of iodinated salicylic acid 1

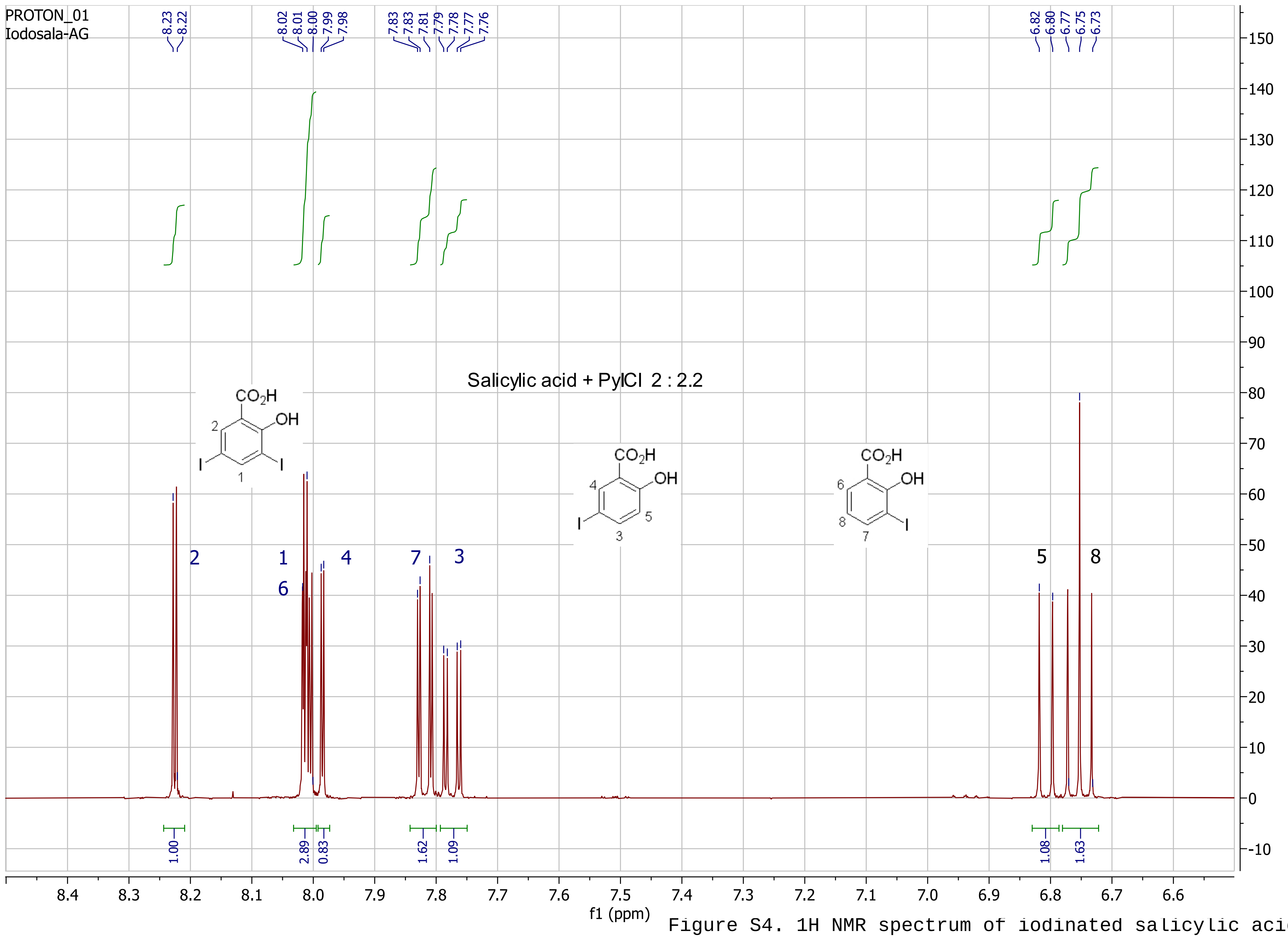

Figure S4. 1H NMR spectrum of iodinated salicylic acid 2
